# Dr. Niels R. Finsen

**Published:** 1904-10

**Authors:** 


Dr. Niels R. Finsen, of Copenhagen, died September 24, 1904, aged 43 years. He was a pioneer in photography and established at Copenhagen "Finsen's Medical Light Institute" for the treatment of lupus and other skin diseases. He was an indefatiguable worker despite an incurable malady, and left a name and fame that will last during the centuries. His funeral was attended by two kings and many other officials of high rank.

